# Large-scale exome array summary statistics resources for glycemic traits to aid effector gene prioritization

**DOI:** 10.12688/wellcomeopenres.18754.1

**Published:** 2023-10-20

**Authors:** Sara M. Willems, Natasha H. J. Ng, Juan Fernandez, Rebecca S. Fine, Eleanor Wheeler, Jennifer Wessel, Hidetoshi Kitajima, Gaelle Marenne, Xueling Sim, Hanieh Yaghootkar, Shuai Wang, Sai Chen, Yuning Chen, Yii-Der Ida Chen, Niels Grarup, Ruifang Li-Gao, Tibor V. Varga, Jennifer L. Asimit, Shuang Feng, Rona J. Strawbridge, Erica L. Kleinbrink, Tarunveer S. Ahluwalia, Ping An, Emil V. Appel, Dan E. Arking, Juha Auvinen, Lawrence F. Bielak, Nathan A. Bihlmeyer, Jette Bork-Jensen, Jennifer A. Brody, Archie Campbell, Audrey Y. Chu, Gail Davies, Ayse Demirkan, James S. Floyd, Franco Giulianini, Xiuqing Guo, Stefan Gustafsson, Anne U. Jackson, Johanna Jakobsdottir, Marjo-Riitta Järvelin, Richard A. Jensen, Stavroula Kanoni, Sirkka Keinanen-Kiukaanniemi, Man Li, Yingchang Lu, Jian'an Luan, Alisa K. Manning, Jonathan Marten, Karina Meidtner, Dennis O. Mook-Kanamori, Taulant Muka, Giorgio Pistis, Bram Prins, Kenneth M. Rice, Serena Sanna, Albert Vernon Smith, Jennifer A. Smith, Lorraine Southam, Heather M. Stringham, Vinicius Tragante, Sander W. van der Laan, Helen R. Warren, Jie Yao, Andrianos M. Yiorkas, Weihua Zhang, Wei Zhao, Mariaelisa Graff, Heather M. Highland, Anne E. Justice, Eirini Marouli, Carolina Medina-Gomez, Saima Afaq, Wesam A. Alhejily, Najaf Amin, Folkert W. Asselbergs, Lori L. Bonnycastle, Michiel L. Bots, Ivan Brandslund, Ji Chen, John Danesh, Renée de Mutsert, Abbas Dehghan, Tapani Ebeling, Paul Elliott, EPIC-Interact Consortium, Aliki-Eleni Farmaki, Jessica D. Faul, Paul W. Franks, Steve Franks, Andreas Fritsche, Anette P. Gjesing, Mark O. Goodarzi, Vilmundur Gudnason, Göran Hallmans, Tamara B. Harris, Karl-Heinz Herzig, Marie-France Hivert, Torben Jørgensen, Marit E. Jørgensen, Pekka Jousilahti, Eero Kajantie, Maria Karaleftheri, Sharon L.R. Kardia, Leena Kinnunen, Heikki A. Koistinen, Pirjo Komulainen, Peter Kovacs, Johanna Kuusisto, Markku Laakso, Leslie A. Lange, Lenore J. Launer, Aaron Leong, Jaana Lindström, Jocelyn E. Manning Fox, Satu Männistö, Nisa M. Maruthur, Leena Moilanen, Antonella Mulas, Mike A. Nalls, Matthew Neville, James S. Pankow, Alison Pattie, Eva R.B. Petersen, Hannu Puolijoki, Asif Rasheed, Paul Redmond, Frida Renström, Michael Roden, Danish Saleheen, Juha Saltevo, Kai Savonen, Sylvain Sebert, Tea Skaaby, Kerrin S. Small, Alena Stančáková, Jakob Stokholm, Konstantin Strauch, E-Shyong Tai, Kent D. Taylor, Betina H. Thuesen, Anke Tönjes, Emmanouil Tsafantakis, Tiinamaija Tuomi, Jaakko Tuomilehto, Understanding Society Scientific Group, Matti Uusitupa, Marja Vääräsmäki, Ilonca Vaartjes, Magdalena Zoledziewska, Goncalo Abecasis, Beverley Balkau, Hans Bisgaard, Alexandra I. Blakemore, Matthias Blüher, Heiner Boeing, Eric Boerwinkle, Klaus Bønnelykke, Erwin P. Bottinger, Mark J. Caulfield, John C. Chambers, Daniel I. Chasman, Ching-Yu Cheng, Francis S. Collins, Josef Coresh, Francesco Cucca, Gert J. de Borst, Ian J. Deary, George Dedoussis, Panos Deloukas, Hester M. den Ruijter, Josée Dupuis, Michele K. Evans, Ele Ferrannini, Oscar H. Franco, Harald Grallert, Torben Hansen, Andrew T. Hattersley, Caroline Hayward, Joel N. Hirschhorn, Arfan Ikram, Erik Ingelsson, Fredrik Karpe, Kay-Tee Kaw, Wieland Kiess, Jaspal S. Kooner, Antje Körner, Timo Lakka, Claudia Langenberg, Lars Lind, Cecilia M. Lindgren, Allan Linneberg, Leonard Lipovich, Ching-Ti Liu, Jun Liu, Yongmei Liu, Ruth J.F. Loos, Patrick E. MacDonald, Karen L. Mohlke, Andrew D. Morris, Patricia B. Munroe, Alison Murray, Sandosh Padmanabhan, Colin N. A . Palmer, Gerard Pasterkamp, Oluf Pedersen, Patricia A. Peyser, Ozren Polasek, David Porteous, Michael A. Province, Bruce M. Psaty, Rainer Rauramaa, Paul M. Ridker, Olov Rolandsson, Patrik Rorsman, Frits R. Rosendaal, Igor Rudan, Veikko Salomaa, Matthias B. Schulze, Robert Sladek, Blair H. Smith, Timothy D. Spector, John M. Starr, Michael Stumvoll, Cornelia M. van Duijn, Mark Walker, Nick J. Wareham, David R. Weir, James G. Wilson, Tien Yin Wong, Eleftheria Zeggini, Alan B. Zonderman, Jerome I. Rotter, Andrew P. Morris, Michael Boehnke, Jose C. Florez, Mark I. McCarthy, James B. Meigs, Anubha Mahajan, Robert A. Scott, Anna L. Gloyn, Inês Barroso

**Affiliations:** 1 MRC Epidemiology Unit, University of Cambridge School of Clinical Medicine, Institute of Metabolic Science, Cambridge Biomedical Campus, Cambridge, CB2 0QQ, UK; 2 General Medicine Center, Saarland University Faculty of Medicine, Homburg, 66421, Germany; 3 Oxford Centre for Diabetes, Endocrinology and Metabolism, University of Oxford, Oxford, OX3 7LE, UK; 4 Stem Cells and Diabetes Laboratory, Institute of Molecular and Cell Biology, Agency for Science, Technology and Research (A*STAR), Singapore, 138673, Singapore; 5 Wellcome Centre for Human Genetics, University of Oxford, Oxford, OX3 7BN, UK; 6 Department of Genetics, Harvard Medical School, Boston, MA, 02115, USA; 7 Division of Endocrinology and Center for Basic and Translational Obesity Research, Boston Children's Hospital, Boston, MA, 02115, USA; 8 Broad Institute of MIT and Harvard, Cambridge, MA, 02142, USA; 9 Current address: Vertex Pharmaceuticals Incorporated, 50 Northern Avenue, Boston, MA, 02210, USA; 10 Department of Human Genetics, Wellcome Sanger Institute, Genome Campus, Hinxton, Cambridge, CB10 1SA, UK; 11 Departments of Epidemiology & Medicine, Schools of Public Health & Medicine, Indiana University, Indianapolis, IN, 46202, USA; 12 Diabetes Translational Research Center, Indiana University School of Medicine, Indianapolis, IN, 46202, USA; 13 General Medicine Division, Massachusetts General Hospital, Boston, MA, USA; 14 Saw Swee Hock School of Public Health, National University Health System, National University of Singapore, Singapore, 117549, Singapore; 15 Department of Biostatistics and Center for Statistical Genetics, University of Michigan, Ann Arbor, MI, 48109, USA; 16 Genetics of Complex Traits, University of Exeter Medical School, University of Exeter, Exeter, EX2 5DW, UK; 17 Department of Biostatistics, Boston University School of Public Health, Boston, MA, USA; 18 The Institute for Translational Genomics and Population Sciences, Department of Pediatrics, The Lundquist Institute for Biomedical Innovation at Harbor-UCLA Medical Center, Torrance, CA, 90502, USA; 19 Novo Nordisk Foundation Center for Basic Metabolic Research, Faculty of Health and Medical Sciences, University of Copenhagen, Copenhagen, 2200, Denmark; 20 Department of Clinical Epidemiology, Leiden University Medical Center, Leiden, 2333 ZA, The Netherlands; 21 Department of Clinical Sciences, Genetic and Molecular Epidemiology Unit, Lund University, Malmö, SE-205 02, Sweden; 22 MRC Biostatistics Unit, University of Cambridge, Cambridge, CB2 0SR, UK; 23 Department of Biostatistics, University of Michigan School of Public Health, Ann Arbor, MI, USA; 24 Mental Health and Wellbeing, School of Health and Wellbeing, College of Medical, Veterinary and Life Sciences, University of Glasgow, Glasgow, G12 8RZ, UK; 25 Cardiovascular Medicine Unit, Department of Medicine Solna, Karolinska Institute, Stockholm, 171 76, Sweden; 26 Quantitative Life Sciences, McGill University, Montreal, Quebec, Canada; 27 Center for Molecular Medicine and Genetics, Wayne State University, Detroit, MI, 48201-1928, USA; 28 COPSAC, Copenhagen Prospective Studies on Asthma in Childhood, Herlev and Gentofte Hospital, University of Copenhagen, Copenhagen, Denmark; 29 Steno Diabetes Center Copenhagen, Gentofte, 2820, Denmark; 30 Department of Genetics, Division of Statistical Genomics, Washington University School of Medicine, St. Louis, Missouri, 63108, USA; 31 McKusick-Nathans Institute, Department of Genetic Medicine, Johns Hopkins University School of Medicine, Baltimore, MD, USA; 32 Center for Life Course Health Research, University of Oulu, Oulu, 90014, Finland; 33 Unit of Primary Care, Oulu University Hospital, Oulu, Finland; 34 Department of Epidemiology, School of Public Health, University of Michigan, Ann Arbor, MI, 48109, USA; 35 McKusick-Nathans Institute of Genetic Medicine, Johns Hopkins University School of Medicine, Baltimore, MD, USA; 36 Cardiovascular Health Research Unit, University of Washington, Seattle, WA, 98195, USA; 37 Department of Medicine, University of Washington, Seattle, WA, USA; 38 Centre for Genomic and Experimental Medicine, Institute of Genetics and Cancer, University of Edinburgh, Edinburgh, EH4 2XU, UK; 39 Division of Preventive Medicine, Brigham and Women's Hospital, Boston, MA, 02215, USA; 40 Centre for Cognitive Ageing and Cognitive Epidemiology, University of Edinburgh, Edinburgh, EH8 9JZ, UK; 41 Department of Psychology, University of Edinburgh, Edinburgh, EH8 9JZ, UK; 42 Department of Epidemiology, Erasmus University Medical Center, Rotterdam, 3015 GE, The Netherlands; 43 Department of Medical Sciences, Molecular Epidemiology and Science for Life Laboratory, Uppsala University, Uppsala, 75237, Sweden; 44 Icelandic Heart Association, Kopavogur, Iceland; 45 Department of Epidemiology and Biostatistics, MRC-PHE Centre for Environment & Health, School of Public Health, Imperial College London, London, W2 1PG, UK; 46 Biocenter Oulu, University of Oulu, Oulu, Finland; 47 William Harvey Research Institute, Barts and The London School of Medicine and Dentistry, Queen Mary University of London, London, UK; 48 Faculty of Medicine, Center for Life Course Health Research, University of Oulu, Oulu, Finland; 49 MRC and Unit of Primary Care, Oulu University Hospital, Oulu, Finland; 50 Division of Nephrology, Internal Medicine, School of Medicine, University of Utah, Salt Lake City, USA; 51 Department of Epidemiology, Johns Hopkins Bloomberg School of Public Health, Baltimore, Maryland, USA; 52 The Charles Bronfman Institute for Personalized Medicine, The Icahn School of Medicine at Mount Sinai, New York, NY, 10069, USA; 53 Department of Medicine, Division of Genetic Medicine, Vanderbilt Genetics Institute, Vanderbilt University School of Medicine, Nashville, TN, 37203, USA; 54 Center for Human Genetics Research, Massachusetts General Hospital, Boston, MA, 02114, USA; 55 Department of Medicine, Harvard Medical School, Boston, MA, USA; 56 Medical Research Council Human Genetics Unit, Institute of Genetics and Cancer, University of Edinburgh, Edinburgh, EH4 2XU, UK; 57 Department of Molecular Epidemiology, German Institute of Human Nutrition Potsdam-Rehbruecke (DIfE), Nuthetal, 14558, Germany; 58 German Center for Diabetes Research (DZD), München-Neuherberg, 85764, Germany; 59 Department of Public Health and Primary Care, Leiden University Medical Center, Leiden, 2333 ZA, The Netherlands; 60 Institute of Social and Preventive Medicine, University of Bern, Bern, Switzerland; 61 Italian National Research Council, Institute of Genetics and Biomedic Research, Cittadella Universitaria, Monserrato, 09042, Italy; 62 Center for Statistical Genetics, University of Michigan, Ann Arbor, MI, 48109, USA; 63 Department of Biostatistics, University of Washington, Seattle, WA, USA; 64 University Medical Center Groningen, Department of Genetics, University of Groningen, Groningen, 9700 RB, The Netherlands; 65 Faculty of Medicine, University of Iceland, Reykjavik, Iceland; 66 Survey Research Center, Institute for Social Research, University of Michigan, Ann Arbor, MI, 48104, USA; 67 Institute of Translational Genomics, Helmholtz Zentrum München, German Research Center for Environmental Health, Neuherberg, Germany; 68 Department of Cardiology, Division Heart & Lungs, University Medical Center Utrecht, Utrecht University, Utrecht, 3584CX, The Netherlands; 69 Central Diagnostics Laboratory, Division Laboratories, Pharmacy, and Biomedical genetics, University Medical Center Utrecht, Utrecht University, Utrecht, The Netherlands; 70 Barts Cardiovascular Research Unit, Barts and The London School of Medicine & Dentistry, Queen Mary University, London, EC1M 6BQ, UK; 71 Section of Investigative Medicine, Department of Medicine, Imperial College London, London, W12 0NN, UK; 72 Department of Life Sciences, Brunel University London, London, UB8 3PH, UK; 73 Department of Epidemiology and Biostatistics, Imperial College London, London, W2 1PG, UK; 74 Ealing Hospital, London North West Healthcare NHS Trust, Middlesex, UB1 3HW, UK; 75 Department of Epidemiology, University of North Carolina at Chapel Hill, Chapel Hill, NC, 27514, USA; 76 Human Genetics Center, The University of Texas School of Public Health; The University of Texas Graduate School of Biomedical Sciences at Houston;, The University of Texas Health Science Center at Houston, Houston, TX, 77030, USA; 77 Department of Internal Medicine, Erasmus University Medical Center, Rotterdam, 3015 GE, The Netherlands; 78 Department of Medicine, King Abdulaziz University, Jeddah, 21589, Saudi Arabia; 79 Amsterdam University Medical Centers, Department of Cardiology, University of Amsterdam, Amsterdam, The Netherlands; 80 Health Data Research UK and Institute of Health Informatics, University College London, London, UK; 81 Center for Precision Health Research, National Human Genome Research Institute, NIH, Bethesda, MD, 20892, USA; 82 Center for Circulatory Health, University Medical Center Utrecht, Utrecht, 3508GA, The Netherlands; 83 Department of Clinical Biochemistry, Lillebaelt Hospital Vejle, Vejle, 7100, Denmark; 84 Institute of Regional Health Research, University of Southern Denmark, Odense, 5000, Denmark; 85 Department of Public Health and Primary Care, University of Cambridge, Cambridge, CB18RN, UK; 86 UK Dementia Research Institute, Imperial College London, London, UK; 87 Oulu University Hospital, Oulu, 90220, Finland; 88 Imperial College NIHR Biomedical Research Centre, London, UK; 89 Health Data Research UK, Imperial College London, London, UK; 90 Department of Nutrition and Dietetics, School of Health Science and Education, Harokopio University, Athens, 17671, Greece; 91 Department of Population Science and Experimental Medicine, Institute of Cardiovascular Science, University College London, London, UK; 92 Department of Nutrition, Harvard School of Public Health, Boston, MA, USA; 93 Institute of Reproductive and Developmental Biology, Imperial College London, London, W12 0NN, UK; 94 Department of Internal Medicine, Division of Endocrinology, Diabetology, Vascular Medicine, Nephrology, and Clinical Chemistry, University Hospital of Tübingen, Tübingen, Germany; 95 Division of Endocrinology, Diabetes and Metabolism, Cedars-Sinai Medical Center, Los Angeles, CA, 90048, USA; 96 Department of Biobank Research, Umeå University, Umeå, SE-901 87, Sweden; 97 Institute of Biomedicine and Biocenter of Oulu, Faculty of Medicine, Medical Research Center Oulu and Oulu University Hospital, Oulu, Finland; 98 Department of Gastroenterology and Metabolism, Poznan University of Medical Sciences, Poznan, 60-572, Poland; 99 Department of Population Medicine, Harvard Medical School, Harvard Pilgrim Health Care Institute, Boston, MA, USA; 100 Diabetes Unit, Department of Medicine, Massachusetts General Hospital, Boston, MA, USA; 101 Center for Clinical Research and Prevention, Bispebjerg and Frederiksberg Hospital, Frederiksberg, 2000, Denmark; 102 Department of Public Health, Faculty of Health and Medical Sciences, University of Copenhagen, Copenhagen, 2200, Denmark; 103 Faculty of Medicine, University of Aalborg, Aalborg, 9100, Denmark; 104 National Institute of Public Health, Southern Denmark University, Odense, 5000, Denmark; 105 Department of Public Health and Welfare, Finnish Institute for Health and Welfare, Helsinki, FI-00271, Finland; 106 PEDEGO Research Unit, MRC Oulu, Oulu University Hospital and University of Oulu, Oulu, Finland; 107 Department of Clinical and Molecular Medicine, Norwegian University of Science and Technology, Trondheim, Norway; 108 Children’s Hospital, Helsinki University Hospital and University of Helsinki, Helsinki, Finland; 109 Echinos Medical Centre, Echinos, Greece; 110 University of Helsinki and Department of Medicine, Helsinki University Hospital, Helsinki, FI-00029, Finland; 111 Minerva Foundation Institute for Medical Research, Biomedicum 2U Helsinki, Helsinki, FI-00290, Finland; 112 Foundation for Research in Health Exercise and Nutrition, Kuopio Research Institute of Exercise Medicine, Kuopio, 70100, Finland; 113 Integrated Research and Treatment (IFB) Center Adiposity Diseases, University of Leipzig, Leipzig, 04103, Germany; 114 Medical Department III – Endocrinology, Nephrology, Rheumatology, University of Leipzig Medical Center, Leipzig, 04103, Germany; 115 Institute of Clinical Medicine, Internal Medicine, University of Eastern Finland, Kuopio, 70210, Finland; 116 Department of Medicine, Division of Bioinformatics and Personalized Medicine, University of Colorado Denver, Denver, CO, USA; 117 Laboratory of Epidemiology and Population Sciences, National Institute on Aging, National Institutes of Health, Baltimore, MD, 21224, USA; 118 Division of General Internal Medicine, Massachusetts General Hospital, Department of Medicine, Harvard Medical School, Boston, MA, USA; 119 Alberta Diabetes Institute IsletCore, University of Alberta, Edmonton, T6G 2E1, Canada; 120 Department of Pharmacology, University of Alberta, Edmonton, T6G 2E1, Canada; 121 Department of Medicine, Division of General Internal Medicine, Johns Hopkins University School of Medicine, Baltimore, MD, USA; 122 Welch Center for Prevention, Epidemiology, and Clinical Research, Johns Hopkins University, Baltimore, MD, USA; 123 Kuopio University Hospital, Kuopio, 70210, Finland; 124 Dipartimento di Scienze Biomediche, Università degli Studi di Sassari, Sassari, 07100, Italy; 125 Laboratory of Neurogenetics, National Institute on Aging, Bethesda, MD, 20892, USA; 126 Data Tecnica International LLC, Glen Echo, MD, 20812, USA; 127 Division of Epidemiology and Community Health, School of Public Health, University of Minnesota, Minneapolis, MN, 55455, USA; 128 South Ostobothnia Central Hospital, Seinajoki, 60220, Finland; 129 Center for Non-Communicable Diseases, Karachi, Pakistan; 130 Institute for Clinical Diabetology, German Diabetes Center, Leibniz Institute for Diabetes Research at Heinrich Heine University Düsseldorf, Düsseldorf, Germany; 131 Division of Endocrinology and Diabetology, Medical Faculty, University Hospital Düsseldorf, Düsseldorf, Germany; 132 Department of Biostatistics and Epidemiology, University of Pennsylvania, 19104, USA; 133 Central Finland Central Hospital, Jyvaskyla, 40620, Finland; 134 Department of Clinical Physiology and Nuclear Medicine, Kuopio University Hospital, Kuopio, 70029, Finland; 135 Department of Twin Research and Genetic Epidemiology, King's College London, London, SE1 7EH, UK; 136 Institute of Genetic Epidemiology, Helmholtz Center Munich, German Research Center for Environmental Health, German Center for Diabetes Research (DZD e.V.), Neuherberg, Germany; 137 Department of Medicine, Yong Loo Lin School of Medicine, National University of Singapore, Singapore, 119228, Singapore; 138 Duke-NUS Medical School, Singapore, 169857, Singapore; 139 Department of Medicine, University of Leipzig, Leipzig, 04103, Germany; 140 Anogia Medical Centre, Anogia, Greece; 141 Folkhälsan Research Centre, Helsinki, Finland; 142 Department of Endocrinology, Helsinki University Central Hospital, Helsinki, Finland; 143 Institute for Molecular Medicine Finland FIMM, University of Helsinki, Helsinki, Finland; 144 Department of Clinical Sciences, Diabetes and Endocrinology, Lund University Diabetes Centre, Malmö, Sweden; 145 Department of Public Health, University of Helsinki, Helsinki, Finland; 146 Saudi Diabetes Research Group, King Abdulaziz University, Jeddah, 21589, Saudi Arabia; 147 Department of Public Health and Clinical Nutrition, University of Eastern Finland, Kuopio, 70210, Finland; 148 Department of Welfare, Children, Adolescents and Families Unit, National Institute for Health and Welfare, Oulu, Finland; 149 INSERM U1018, Centre de recherche en Épidémiologie et Santé des Populations (CESP), Villejuif, France; 150 Helmholtz Institute for Metabolic, Obesity and Vascular Research (HI-MAG), Helmholtz Zentrum München, University of Leipzig and University Hospital Leipzig, Leipzig, Germany; 151 Department of Epidemiology, German Institute of Human Nutrition Potsdam-Rehbrücke (DIfE), Nuthetal, 14558, Germany; 152 The Human Genetics Center and Institute of Molecular Medicine, University of Texas Health Science Center, Houston, Texas, 77030, USA; 153 Imperial College Healthcare NHS Trust, London, W12 0HS, UK; 154 Harvard School of Medicine, Boston, MA, USA; 155 Division of Genetics, Brigham and Women's Hospital and Harvard Medical School, Boston, MA, USA; 156 Singapore Eye Research Institute, Singapore National Eye Centre, Singapore, 169856, Singapore; 157 Ophthalmology & Visual Sciences Academic Clinical Program (Eye ACP), Duke-NUS Medical School, Singapore, 169857, Singapore; 158 Department of Ophthalmology, Yong Loo Lin School of Medicine, National University of Singapore, Singapore, 119228, Singapore; 159 Department of Vascular Surgery, Division of Surgical Specialties, University Medical Center Utrecht, Utrecht, 3584 CX, The Netherlands; 160 Princess Al-Jawhara Al-Brahim Centre of Excellence in Research of Hereditary Disorders (PACER-HD), King Abdulaziz University, Jeddah, Saudi Arabia; 161 Experimental Cardiology Laboratory, Division Heart and Lungs, University Medical Center Utrecht, Utrecht University, Utrecht, 3584 CX, The Netherlands; 162 Department of Epidemiology, Biostatistics and Occupational Health, McGill University, Montreal, Quebec, Canada; 163 CNR Institute of Clinical Physiology, Department of Clinical & Experimental Medicine, University of Pisa, Pisa, Italy; 164 Institute of Epidemiology II, Research Unit of Molecular Epidemiology, Helmholtz Zentrum München, Munich, Germany; 165 Faculty of Health Sciences, University of Southern Denmark, Odense, 5000, Denmark; 166 University of Exeter Medical School, University of Exeter, Exeter, EX2 5DW, UK; 167 Departments of Pediatrics and Genetics, Harvard Medical School, Boston, MA, 02115, USA; 168 Department of Medicine, Division of Cardiovascular Medicine, Stanford University School of Medicine, Stanford, CA, 94305, USA; 169 Stanford Cardiovascular Institute, Stanford University, Stanford, CA, 94305, USA; 170 Stanford Diabetes Research Center, Stanford University, Stanford, 94305, USA; 171 Oxford NIHR Biomedical Research Centre, Churchill Hospital, Oxford, OX3 7LE, UK; 172 Department of Public Health and Primary Care, Institute of Public Health, University of Cambridge, Cambridge, CB1 8RN, UK; 173 Pediatric Research Center, Department of Women & Child Health, University of Leipzig, Leipzig, Germany; 174 National Heart and Lung Institute, Imperial College London, London, W12 0NN, UK; 175 Institute of Biomedicine, School of Medicine, University of Eastern Finland, Kuopio, 70211, Finland; 176 Department of Medical Sciences, Molecular Epidemiology; EpiHealth, Uppsala University, Uppsala, 75185, Sweden; 177 The Big Data Institute, Li Ka Shing Centre for Health Information and Discovery, University of Oxford, Oxford, OX3 7BN, UK; 178 Department of Clinical Medicine, Faculty of Health and Medical Sciences, University of Copenhagen, Copenhagen, 2200, Denmark; 179 Department of Neurology, Wayne State University School of Medicine, Detroit, MI, USA; 180 Department of Epidemiology & Prevention, Division of Public Health Sciences, Wake Forest University, Winston-Salem, NC, 27157, USA; 181 The Mindich Child Health and Development Institute, The Icahn School of Medicine at Mount Sinai, New York, NY, 10069, USA; 182 Department of Genetics, University of North Carolina, Chapel Hill, NC, 27599, USA; 183 Usher Institute of Population Health Sciences and Informatics, University of Edinburgh, Edinburgh, EH16 4UX, UK; 184 Aberdeen Biomedical Imaging Centre, University of Aberdeen, Foresterhill Health Campus, Aberdeen, AB25 2ZD, UK; 185 British Heart Foundation Glasgow Cardiovascular Research Centre, Institute of Cardiovascular and Medical Sciences, College of Medical, Veterinary and Life Sciences, University of Glasgow, Glasgow, G12 8TA, UK; 186 Division of Population Health and Genomics, School of Medicine, University of Dundee, Dundee, DD2 4BF, UK; 187 Laboratory of Clinical Chemistry and Hematology, University Medical Center Utrecht, Utrecht, 3584 CX, The Netherlands; 188 Faculty of Medicine, University of Split, Split, Croatia; 189 Departments of Epidemiology, Health Systems and Population Health, University of Washington, Seattle, Seattle, WA, USA; 190 Division of Cardiovascular Medicine, Brigham and Women's Hospital, Boston, MA, 02115, USA; 191 Department of Public Health & Clinical Medicine, Section for Family Medicine, Umeå University, Umeå, SE-901 85, Sweden; 192 Department of Medicine, McGill University, Montreal, Quebec, H4A 3J1, Canada; 193 Department of Human Genetics, McGill University, Montreal, Quebec, H3A 1B1, Canada; 194 Alzheimer Scotland Dementia Research Centre, University of Edinburgh, Edinburgh, EH8 9JZ, UK; 195 Institute of Cellular Medicine, The Medical School, Newcastle University, Newcastle, NE2 4HH, UK; 196 Department of Physiology and Biophysics, University of Mississippi Medical Center, Jackson, MS, USA; 197 Technical University of Munich (TUM) and Klinikum Rechts der Isar, TUM School of Medicine, Munich, Germany; 198 Centre for Genetics and Genomics Versus Arthritis, Centre for Musculoskeletal Research, University of Manchester, Manchester, UK; 199 Diabetes Unit and Center for Genomic Medicine, Massachusetts General Hospital, Boston, MA, USA; 200 Programs in Metabolism and Medical & Population Genetics, Broad Institute, Cambridge, MA, USA; 201 Current address: Genentech, South San Francisco, CA, 94080, USA; 202 Division of Endocrinology, Department of Pediatrics, Stanford School of Medicine, Stanford, CA, USA; 203 Exeter Centre of Excellence in Diabetes (EXCEED), University of Exeter Medical School, Exeter, UK

**Keywords:** exome chip, glycaemic traits, genetic discovery, effector genes, summary statistics resources

## Abstract

**Background:**

Genome-wide association studies for glycemic traits have identified hundreds of loci associated with these biomarkers of glucose homeostasis. Despite this success, the challenge remains to link variant associations to genes, and underlying biological pathways.

**Methods:**

To identify coding variant associations which may pinpoint effector genes at both novel and previously established genome-wide association loci, we performed meta-analyses of exome-array studies for four glycemic traits: glycated hemoglobin (HbA1c, up to 144,060 participants), fasting glucose (FG, up to 129,665 participants), fasting insulin (FI, up to 104,140) and 2hr glucose post-oral glucose challenge (2hGlu, up to 57,878). In addition, we performed network and pathway analyses.

**Results:**

Single-variant and gene-based association analyses identified coding variant associations at more than 60 genes, which when combined with other datasets may be useful to nominate effector genes. Network and pathway analyses identified pathways related to insulin secretion, zinc transport and fatty acid metabolism. HbA1c associations were strongly enriched in pathways related to blood cell biology.

**Conclusions:**

Our results provided novel glycemic trait associations and highlighted pathways implicated in glycemic regulation. Exome-array summary statistic results are being made available to the scientific community to enable further discoveries.

## Introduction

Genome-wide association studies (GWAS) have identified hundreds of loci associated with glycemic traits and type 2 diabetes (T2D) risk ^1– 3^. Despite this tremendous success, the challenge remains to link the often lead non-coding variants with effector genes and mechanism of action. To complement these approaches, exome array studies ^4, 5^ and more recently, whole-exome sequencing approaches have focused on coding variant associations ^6– 9^. These can be helpful to pinpoint potential effector genes for downstream functional studies. Here, we provide exome-array GWAS meta-analysis results for glycated hemoglobin (HbA1c, up to 144,060 participants), fasting glucose (FG, up to 129,665 participants), fasting insulin (FI, up to 104,140) and 2hr glucose post-oral glucose challenge (2hGlu, up to 57,878). Most of the data are from self-reported and genetically clustered European ancestry individuals (85%), with the remaining participants being of African American (6%), South Asian (5%), East Asian (2%) and Hispanic ancestry (2%). We identify single coding variant and gene-based associations to prioritize likely effector genes, and additionally perform pathway analyses to highlight relevant gene sets regulating each glycemic trait. Summary statistics from these analyses are publicly available through our website ( www.magicinvestigators.org), as well as through the GWAS catalog ( https://www.ebi.ac.uk/gwas/summary-statistics, study accessions GCST90256400 - GCST90256420) ^10^.

## Methods

### Study design, cohorts, phenotypes and genotypes

MAGIC (Meta-Analysis of Glucose and Insulin-related traits Consortium) was established to focus on the genetic analysis of glycemic traits in individuals without diabetes. In this MAGIC effort, individuals without diabetes of self-reported and genetically clustered European (85%), African American (6%), South Asian (5%), East Asian (2%) and Hispanic (2%) ancestry from up to 64 cohorts participated. Sample sizes were up to 144,060 for HbA1c, 129,665 for FG, 104,140 for FI and 57,878 for 2hGlu. Participating cohorts and their characteristics are detailed in Supplementary Table S1 ^11^. Each cohort obtained ethical approval and written informed consent.

### Phenotypes

Studied outcomes were FG (mmol/L), Ln-transformed FI (pmol/L), 2hGlu (mmol/L) and HbA1c (% of hemoglobin). Glycemic measurements are described in detail for each contributing cohort in Supplementary Table S1 ^11^. Individuals with diagnosed or treated diabetes, or those with diabetes based on FG (≥7 mmol/L), 2hGlu (≥11.1 mmol/L) and/or HbA1c (≥6.5%) were excluded from analyses.

### Genotyping and QC

The Illumina HumanExome BeadChip is a genotyping array containing variants that have been observed in sequencing data of ~12,000 individuals. Non-synonymous variants seen at least three times across at least two datasets were included on the exome chip. More lenient criteria were used for splice and nonsense variants. Besides the core content of protein-altering variants, the exome chip contains additional variants including common variants identified in GWAS, ancestry informative markers, mitochondrial variants, randomly selected synonymous variants, HLA tag variants and Y chromosome variants. In this study we analyzed association with glycemic traits of 247,470 autosomal and X chromosome variants present on the exome chip. Genotype calling and quality control were performed following protocols developed by the UK Exome Chip or CHARGE consortium ^12^. The exact genotyping array, calling algorithm and QC procedure used by each cohort are depicted in Supplementary Table S1 ^11^.

### Annotation and functional prediction of variants

Annotation of the exome chip variants was performed using the Ensembl Variant Effect Predictor v78 with plugin dbNSFP v2.9 to add *in silico* functional prediction from Polyphen HumDiv, Polyphen HumVar, LRT, Mutation Taster and SIFT (ensembl66 version) ^13, 14^.

### Statistical analyses

***Single variant analyses.*** Individual cohorts ran linear mixed models using the raremetalworker (v 4.13.2) or rvtests (v20140723) software (Supplementary Table S1 ^11^). For each glycemic outcome, analyses were performed using an additive model for the raw and the inverse normal transformed trait. In the manuscript and in all tables and figures effect estimates and standard errors are for the raw trait, while the p-values are from the inverse normal transformed trait analyses. Analyses were adjusted for age, sex, BMI, study-specific number of PCs and other study-specific covariates (Supplementary Table S1 ^11^). Raremetal (v4.13.7 or higher) was used to combine results within and across ancestries by fixed-effect meta-analyses. Variants with *P* <10 ^-4^ for deviation from Hardy-Weinberg equilibrium or with call rate <0.99 in individual cohorts were excluded from meta-analyses. In single variant analyses, the threshold for significance was *P* <2.2×10 ^-7^ for coding variants (stop-gained, stop lost, frameshift, splice donor, splice acceptor, initiator codon, missense, in-frame indel and splice region variants). This *P*-value threshold was based on a Bonferroni correction weighted by the enrichment for complex trait associations among the functional annotation categories ^15, 16^. We performed so called distance-based clumping; significant association signals located more than 500 kb apart were considered to represent distinct loci. Significantly associated variants located more than 500 kb from any variant already found to be associated in published large-scale glycemic trait and T2D GWAS analyses ^1, 3, 17, 18^ were considered novel glycemic trait associations. Gene-based and single-variant analyses results presented in the paper are for the meta-analyses of all ancestries combined, unless mentioned otherwise.

***Gene-based analyses.*** Raremetal (v4.13.7 or higher) was used to perform gene-based burden and sequence kernel association (SKAT) tests. For both burden and SKAT tests, two *in silico* masks for inclusion of variants in the test were used: NSstrict and NSbroad. The NSstrict mask includes predicted protein truncating variants (PTVs, splice donor, splice acceptor, stop gained, frameshift, stop lost or initiator codon variant) OR variants that are missense and predicted to be damaging by five prediction algorithms (SIFT, Polyphen HumDiv, Polyphen HumVar, LRT, MutationTaster). The NSbroad mask additionally includes missense variants predicted to be damaging by at least one of the five prediction algorithms AND that have a MAF <1% in each ancestry group. These MAFs were derived from our single variant HbA1c meta-analyses results (N up to 144,060). Gene-based analyses were performed on genes containing at least two variants fulfilling the mask criteria. The *P*-value threshold for significance in gene-based analyses was 2.5 x 10 ^-6^ (Bonferroni correction for 20,000 genes).

### GeneMANIA network analysis

**Figure 1.**
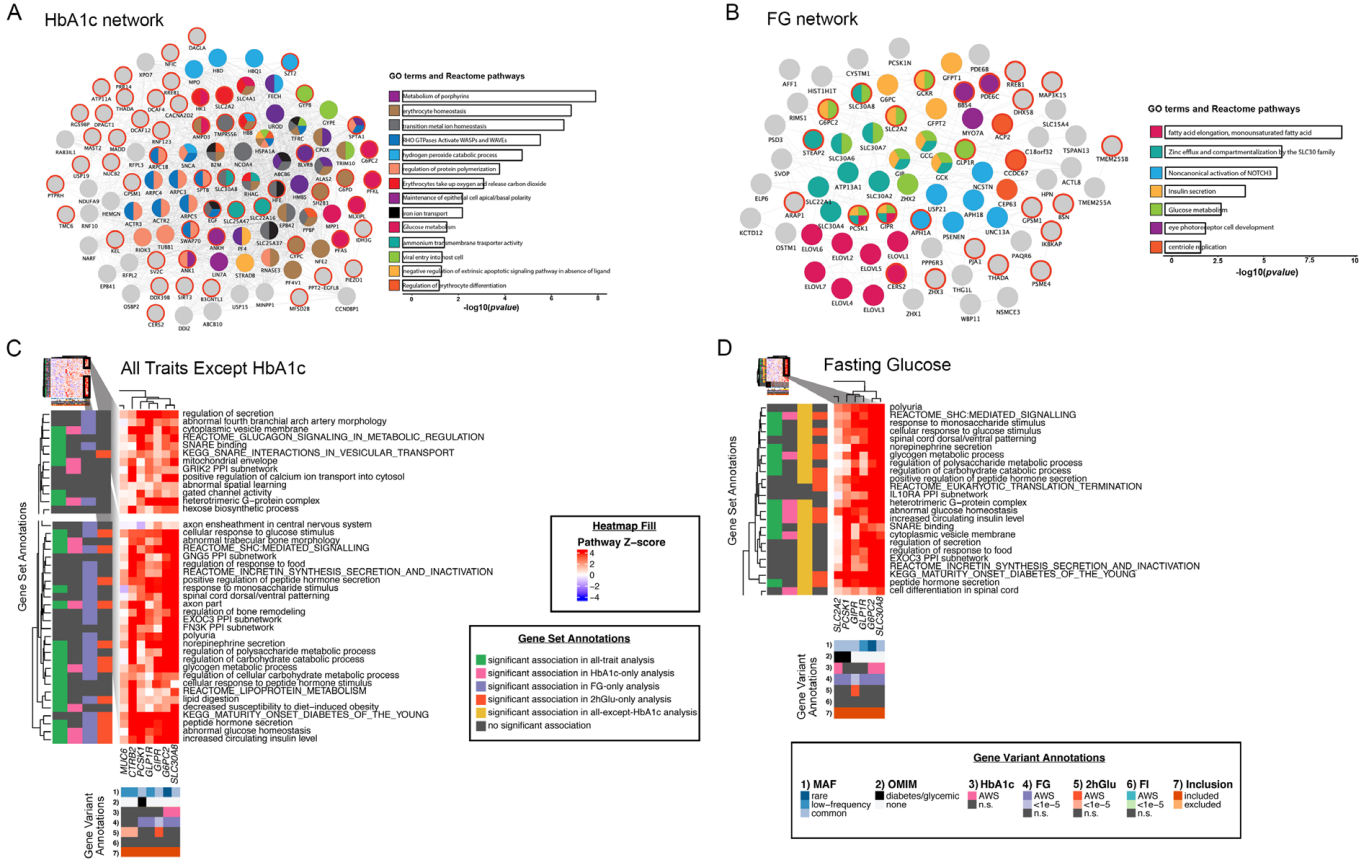
Network and pathway analyses identify relevant gene sets regulating glycemia using two different methods for variant associations with *P* <1 × 10 ^-5^. ( **A–B**) The networks represent composite networks for ( **A**) HbA1c and ( **B**) FG, from the GeneMANIA analysis using genes with variant associations at *P* <1 × 10 ^-5^ for each trait as input. Nodes outlined in red correspond to genes from the input list. Other nodes correspond to related genes based on 50 default databases. Based on the network, GO terms and Reactome pathways that were significantly enriched are depicted. To summarize these results, the most significant term of all calculated terms within the same group is represented. Barplots with the Bonferroni-adjusted -log10(p-values) of the most significant terms within each group are are shown. Each group was assigned a specific color; if a gene is present in more than one term, it is displayed in more than one color. ( **C–D**) Heatmaps showing EC-DEPICT results from analysis of ( **C**) all traits except HbA1c and ( **D**) FG. The columns represent the input genes for the analysis. In ( **C**), these are genes with variant associations of *P* <1 × 10 ^-5^ for FG, FI, and/or 2hGlu, and in ( **D**) these are genes with variant associations of *P* <1 × 10 ^-5^ for FG. Rows in the heatmap represent significant meta-gene sets (FDR <0.05). The color of each square indicates DEPICT’s z-score for membership of that gene in that gene set, where dark red means “very likely a member” and dark blue means “very unlikely a member.” The gene set annotations indicate whether that meta-gene set was significant at FDR <0.05 or not significant (n.s.) for each of the other EC-DEPICT analyses. For heatmap intensity and EC-DEPICT *P*-values, the meta-gene set values are taken from the most significantly enriched member gene set. The gene variant annotations are as follows: (1) the European minor allele frequency (MAF) of the input variant, where rare is MAF <1%, low-frequency is MAF 1–5%, and common is MAF >5%, 2) whether the gene has an Online Mendelian Inheritance in Man (OMIM) annotation as causal for a diabetes/glycemic-relevant syndrome or blood disorder, 3) to 6) whether each variant was significant ( *P* <2 × 10 ^-7^), suggestively significant ( *P* <1 × 10 ^-5^), or not significant in Europeans for each of the four traits, and 7) whether each variant was included in the analysis or excluded by filters (see Methods). AWS: array-wide significant.

For network analyses, we used GeneMANIA (v3.5.1), a network approach that searches many large, publicly available biological datasets to find related genes. These include protein-protein, protein-DNA and genetic interactions, pathways, reactions, gene and protein expression data, protein domains and phenotypic screening profiles. GeneMANIA uses a label propagation algorithm for predicting gene function given the composite functional association network (calculated from the databases selected). The weights needed for the label propagation method to work are selected at the beginning of the process. In our case, and according to the defaults, we weighted the network using linear regression, to make genes in the input list interact as much as possible with each other. We analyzed all loci that had at least one non-synonymous variant with *P* <1 x 10 ^-5^ with any trait, and then mapped the most significant non-synonymous variant at each locus to the gene (input genes). We performed four network analyses: (1) HbA1c-associated variants only, (2) FI-associated variants only, (3) FG-associated variants only, and (4) 2hGlu-associated variants only ( Figure 1, Supplementary Figure S1 ^11^). We selected the 50 default databases to create the composite network, and we allowed the method to find at most 50 genes that are related to our query input list. The resultant networks were investigated to find enriched Gene Ontology (GO) terms and Reactome Pathways. Gene Set Enrichment (GSE) of networks and sub-networks were assessed with ClueGO ^19^ using GO terms and Reactome gene sets ^20^. The enrichment results were grouped using a Cohen’s Kappa score of 0.4, and terms were considered significant with a Bonferroni-adjusted p-value <0.05, provided that there was an overlap of at least three network genes in the relevant GO gene set when calculating GO enrichment. For the pathway selection (Reactome), we set a threshold that the network genes should represent at least 4% of the pathway. These values were applied given the recommended defaults when running ClueGO ^19^. Cohen’s Kappa statistic was used to measure the gene-set similarity of GO terms and Reactome pathways and allowed us to group enriched terms into functional groups to improve visualization of enriched pathways. We used all genes with GO annotations and at least one interaction in our network database as the background set.

### Gene set enrichment analysis (GSEA)

An extension of the GWAS GSEA method DEPICT ^21^, EC-DEPICT ^22, 23^, was used for GSEA. The key feature of EC-DEPICT is the use of “reconstituted” gene sets, which are gene sets collected from many different databases (e.g. canonical pathways, protein-protein interaction networks, and mouse phenotypes) that have been extended based on large-scale microarray co-expression data ^21, 24^.

Six groups of variants were analyzed: (1) HbA1c-associated variants only, (2) FI-associated variants only, (3) FG-associated variants only, (4) 2hGlu-associated variants only, (5) all trait-associated variants, and (6) all trait-associated variants except for HbA1c. For each trait, the associated variants based on the European summary statistics were identified and clumped using a +/- 500 kb window. Then, the most significant nonsynonymous variant for each locus was included in the analysis, with a cut-off of *P* <10 ^-5^. Annotations from the CHARGE consortium were used to assign variants to genes (see **URL**). After GSEA, highly correlated gene sets were grouped by affinity propagation clustering of all 14,462 gene sets ^25^ into “meta-gene sets” using SciKitLearn.clustering.AffinityPropagation version 0.17 ^26^. For all visualizations, the gene set within a meta-gene set with the best enrichment *P*-value was used; heat maps were created with the ComplexHeatmap package in R ^27^.

**URL**: CHARGE Consortium ExomeChip annotation file (v6).

*Method and choice of data for permutations:* We performed the EC-DEPICT analysis as described elsewhere ^22, 23^. All analyses are based on a group of 14,462 “reconstituted” gene sets, which contains a z-score for probability of gene set membership for each gene (for details, see ^21, 24^).

The basic EC-DEPICT method is as follows. We first obtain a list of significant input variants (the most significant nonsynonymous variant per locus) and then map variants to genes based on annotations from the CHARGE consortium (see **URL**). For each gene set, we obtain the gene set membership z-scores for all trait-associated input genes and sum them to generate a test statistic. We then take 2,000 permuted ExomeChip association studies (described in more detail below) and calculate the average permuted test statistic for that gene set, as well as the permuted standard deviation. For each permutation, the number of top genes we take as “input genes” is matched to the actual observed number of input genes. We then calculate (observed test statistic – average permuted test statistic)/(permuted standard deviation) to generate a z-score, which is converted to a p-value via the normal distribution. False discovery rates were calculated by comparing the observed p-values to a permuted *P*-value distribution generated with an additional set of 50 permuted association studies.

The permuted ExomeChip association studies are conducted by (1) generating 2,200 sets of normally distributed phenotypes and (2) using these randomly generated phenotypes to conduct 2,200 association studies with real ExomeChip data. Using these permutations to adjust the observed test statistics corrects for any inherent structure in the data (e.g. that pathways made up of longer genes may be more likely to come up as significant by chance).

For these analyses, we first generated permutations based on ExomeChip data we had used previously for this purpose: 11,899 samples drawn from three cohorts (Malmö Diet and Cancer [MDC], All New Diabetics in Scania [ANDIS], and Scania Diabetes Registry [SDR]). For simplicity, we refer to these cohorts as the “Swedish permutations.”

As part of our GSEA pipeline, we remove input trait-associated variants that are not present in the permuted data to ensure that all variants are appropriately modeled. When using the Swedish permutations, this generally results in removing a substantial fraction of the variants, especially of the very rarest variants (due to the smaller sample size of the Swedish data relative to the data being analyzed). We have previously observed that this filtering can actually improve the GSEA signal, possibly due to more heterogeneous biology or a higher false-positive rate in these very rare variants ^23^. However, in this case, we observed that in performing this filtering, we excluded variants in several known monogenic disease genes, such as *HNF1A* and *SLC2A2*. Therefore, we wished to repeat the analysis with a set of permutations which would allow us to retain these variants. We thus repeated the analysis with a second set of permutations consisting of 152,249 samples from the UK Biobank (referred to as the “UKBB permutations”). The larger sample size in the UKBB permutations means more variants are present and can therefore be included in the analysis.

*Concordance of results from two different sets of permuted distributions across phenotypes:* For completeness, we report the results from the use of both sets of permutations. We note that the results are strongly concordant. The larger number of significant gene sets reported based on the UK Biobank permutations is generally a combination of 1) overall improved power (i.e. more variants are included) and 2) the inclusion of variants in key driver genes absent in the Swedish permutations, encompassing both the monogenic genes mentioned above (e.g. *SLC2A2*) and additional genes with clearly relevant biology (e.g. *SLC30A8*). The results from both sets of permutations are summarized below. For all analyses, “significance” refers to a false discovery rate of <0.05.

*All-trait analysis:* After filtering, 78 input genes were included for the analysis with the UKBB permutations and 60 for the analysis with the Swedish permutations. (Note that the difference in the number of input genes is due to the presence of a larger number of input variants in the UKBB permutations – see above). We found 234 significant gene sets in 86 meta-gene sets based on the UKBB permutations (Supplementary Figure S2 ^11^) and 133 gene sets in 51 meta-gene sets based on the Swedish permutations (Supplementary Figure S3 ^11^). The correlation between the UKBB and Swedish analyses was r = 0.902, *P* <10 ^-300^.

*All-traits-except-HbA1c analysis:* After filtering, 45 input genes were included for the analysis with the UKBB permutations and 33 for the analysis with the Swedish permutations. We found 128 significant gene sets in 53 meta-gene sets based on the UKBB permutations (Supplementary Figure S2 ^11^) and 45 significant gene sets in 18 meta-gene sets based on the Swedish permutations (Supplementary Figure S3 ^11^). The correlation between the UKBB and Swedish analyses was r = 0.882, *P* <10 ^-300^.

*HbA1c-only analysis:* After filtering, 41 input genes were included for the analysis with the UKBB permutations and 33 for the analysis with the Swedish permutations. We found 191 significant gene sets in 73 meta-gene sets based on the UKBB permutations (Supplementary Figure S2 ^11^) and 120 gene sets in 41 meta-gene sets based on the Swedish permutations. (Supplementary Figure S3 ^11^). The correlation between the UKBB and Swedish analyses was r = 0.936, *P* <10 ^-300^.

*FG-only analysis*: After filtering, 26 input genes were included for the analysis with the UKBB permutations and 22 for the analysis with the Swedish permutations. We found 106 significant gene sets in 39 meta-gene sets based on the UKBB permutations (Supplementary Figure S2 ^11^) and 48 significant gene sets in 15 meta-gene sets based on the Swedish permutations (Supplementary Figure S3 ^11^). The correlation between the UKBB and Swedish analyses was r = 0.939, *P* <10 ^-300^.

*2hGlu-only analysis*: After filtering, 12 input genes were included for the analysis with the UKBB permutations and seven for the analysis based on the Swedish permutations. We found 56 significant gene sets in 17 meta-gene sets based on the UKBB permutations (Supplementary Figure S2 ^11^), with no significant gene sets based on the Swedish permutations. The correlation between the UKBB and Swedish analyses was r = 0.787, *P* <10 ^-300^.

*FI-only analysis:* After filtering, 11 input genes were included for the analysis with the UKBB permutations and eight for the analysis with the Swedish permutations. There were no significant gene sets from either analysis. The correlation between the UKBB and Swedish analyses was r = 0.860, *P* <10 ^-300^.

*Visualization*: As in previous work ^22, 23^, we have included all trait-associated variants in the heat maps, even if they were excluded from the analysis (e.g. because they were absent in the permutations or did not have a nonsynonymous annotation in the CHARGE annotation file). This is because we assume that if the genes harboring those variants have strong predicted membership in significantly trait-associated gene sets, they are still good candidates for prioritization. In fact, this may be even stronger evidence in favor of these genes because they did not contribute to the enrichment analysis and therefore their prioritization is independently derived (and provides even more support to the implicated biology).

## Results

### Study design overview

We performed single-variant and gene-based association analyses with FG, FI, HbA1c, and 2hGlu levels on exome-array coding variants in up to 144,060 individuals without diabetes (to exclude any consequence of diabetes treatments or related interventions on these quantitative traits) of European (85%), African-American (6%), South Asian (5%), East Asian (2%), and Hispanic (2%) ancestry from up to 64 cohorts (Supplementary Table S1 ^11^, Methods). We used a linear mixed model to test single-variant associations in each individual cohort and combined results by fixed-effect meta-analyses within and across ancestries. As body mass index (BMI) is a major risk factor for T2D and is correlated with glycemic traits, all analyses were adjusted for BMI to identify loci influencing glycemia independently from their effects on overall adiposity. We have previously demonstrated that collider bias did not significantly affect results with BMI adjustment ^1^. We used distance-based clumping to define distinct loci and considered signals to be novel if they were located more than 500 kb from a variant with an established association with any of the glycemic traits or T2D in large published GWAS (Methods). We considered a coding variant to meet exome-wide significance for association if *P* <2.2 × 10 ^-7^ ^15, 16^ ( Table 1, Methods). To increase power to detect rare variant associations, we additionally performed gene-burden and sequence kernel association (SKAT) tests for gene-level analyses to identify genes with significant evidence of association ( *P* <2.5 × 10 ^-6^) ( Table 2, Methods). Finally, to identify relevant biological pathways enriched in associations with glycemic traits we conducted pathway and network analyses.

**Table 1.** Single-point coding variant associations meeting the significance threshold for coding variants of *P* <2.2 × 10 ^-7^. This table includes all coding variants meeting this threshold, irrespective of whether they fall in completely new loci or in previously-established loci, provided that the association at the established locus was not shown to be due to a non-coding variant (Table S2) or another coding variant at the same locus. Novel loci are highlighted in bold. HbA1c: glycated haemoglobin; FG: fasting glucose; FI: fasting insulin; 2hGlu: 2h glucose; Alleles E/O: effect allele/other allele; EAF: effect allele frequency; Effect (SE): effect size (standard error); *P*: p-value; N: number of samples in the analysis; Novel/previous glycemic trait association: Novel corresponds to a new association result in this study; Locus name of previous association – name used for previously reported locus. ^1^Significant in the European-only analysis in our study. Genes in this table are listed in order of chromosomal position.

Trait	SNP	Gene	Protein Consequence	Alleles E/O	EAF	Effect (SE)	*P*	N	Previous glycemic trait association (if any)	Locus name of previous association
**FG**	**rs1886686**	*WDR78*	**p.G12A**	**G/C**	**0.739**	**0.014 (0.002)**	**2.24×10 ^-11^**	**123558**	**Novel**	
HbA1c	rs267738	*CERS2*	p.E106A	G/T	0.186	-0.01 (0.002)	6.96×10 ^-10^	144043	HbA1c	*CERS2*
HbA1c	rs863362	*OR10X1*	p.W66X	T/C	0.465	0.011 (0.001)	6.76×10 ^-15^	114945	HbA1c	*SPTA1*
HbA1c	rs857725	*SPTA1*	p.K1693Q	G/T	0.262	0.022 (0.001)	1.56×10 ^-50^	143956	HbA1c	*SPTA1*
HbA1c	rs11887523	*MFSD2B*	p.A60T	A/G	0.007	-0.072 (0.01)	1.44×10 ^-12^	122060	HbA1c	*ATAD2B*
FG	rs1260326	*GCKR*	p.L446P	C/T	0.631	0.029 (0.002)	6.36×10 ^-48^	129588	FG, FI, 2hGlu	*GCKR*
FI	rs1260326	*GCKR*	p.L446P	C/T	0.626	0.024 (0.002)	5.55×10 ^-32^	104076	FG, FI, 2hGlu	*GCKR*
2hGlu	rs1260326	*GCKR*	p.L446P	C/T	0.618	-0.069 (0.009)	4.48×10 ^-15^	57813	FG, FI, 2hGlu	*GCKR*
FG	rs35720761	*THADA*	p.C845Y	T/C	0.108	-0.018 (0.003)	4.35×10 ^-9^	129622	T2D, FG	*THADA*
HbA1c	rs35720761	*THADA*	p.C845Y	C/T	0.113	0.014 (0.002)	2.58×10 ^-12^	144001	T2D, FG	*THADA*
FG	rs7578597	*THADA*	p.T897A	C/T	0.106	-0.019 (0.003)	1.99×10 ^-8^	113162	T2D, FG	*THADA*
FI	rs7607980	*COBLL1*	p.N901D	C/T	0.128	-0.032 (0.003)	1.30×10 ^-24^	97817	FI	*COBLL1*
FG	rs2232323	*G6PC2*	p.Y207S	C/A	0.006	-0.129 (0.012)	1.05×10 ^-28^	123981	FG, HbA1c	*G6PC2*
HbA1c	rs2232323	*G6PC2*	p.Y207S	C/A	0.007	-0.053 (0.007)	3.25×10 ^-13^	144038	FG, HbA1c	*G6PC2*
FG	rs146779637	*G6PC2*	p.R283X	T/C	0.002	-0.138 (0.02)	1.78×10 ^-12^	127278	FG, HbA1c	*G6PC2*
HbA1c	rs146779637	*G6PC2*	p.R283X	T/C	0.002	-0.074 (0.012)	4.58×10 ^-10^	141728	FG, HbA1c	*G6PC2*
**FI**	**rs1983210**	*OBSL1*	**p.E1365D**	**G/C**	**0.729**	**0.016 (0.003)**	**8.48×10 ^-10^**	**79767**	**Novel**	
**FI**	**rs3183099**	*OBSL1*	**splice region variant**	**A/G**	**0.226**	**-0.013 (0.002)**	**4.70×10 ^-8^**	**100713**	**Novel**	
FI	rs1801282	*PPARG*	p.P12A	G/C	0.117	-0.031 (0.003)	3.50×10 ^-23^	98631	FI	*PPARG*
HbA1c	rs35726701	*RNF123*	p.K596E	G/A	0.019	0.025 (0.005)	4.19×10 ^-8^	131203	HbA1c	*USP4*
FG	rs5400	*SLC2A2*	p.T110I	A/G	0.161	-0.022 (0.003)	2.14×10 ^-17^	129591	FG, HbA1c	*SLC2A2*
HbA1c	rs5400	*SLC2A2*	p.T110I	A/G	0.153	-0.013 (0.002)	2.27×10 ^-13^	144012	FG, HbA1c	*SLC2A2*
HbA1c ^1^	rs223705	*EGF*	p.M708I	A/G	0.374	-0.007 (0.001)	2.11×10 ^-7^	121204	HbA1c	*EGF*
HbA1c	rs7683365	*GYPB*	p.T48M	A/G	0.312	0.012 (0.002)	1.61×10 ^-8^	45191	HbA1c	*FREM3*
FG	rs146886108	*ANKH*	p.R187Q	T/C	0.004	-0.088 (0.014)	5.67×10 ^-10^	129647	T2D	*ANKH*
**HbA1c**	**rs31244**	*SV2C*	**p.D543N**	**A/G**	**0.083**	**0.012 (0.002)**	**6.05×10 ^-8^**	**144000**	**Novel**	
FG	rs6235	*PCSK1*	p.S690T	G/C	0.264	-0.022 (0.002)	9.22×10 ^-24^	123560	FG	*PCSK1*
2hGlu	rs2549782	*ERAP2*	p.K392N	T/G	0.519	-0.055 (0.009)	6.81×10 ^-10^	57836	2hGlu	*ERAP2*
HbA1c	rs35742417	*RREB1*	p.S1499Y	A/C	0.173	-0.01 (0.002)	3.76×10 ^-9^	143967	FG, T2D	*RREB1*
FG	rs35742417	*RREB1*	p.S1499Y	A/C	0.183	-0.019 (0.002)	1.27×10 ^-16^	129577	FG, T2D	*RREB1*
HbA1c	rs1799945	*HFE*	p.H63D	G/C	0.129	-0.023 (0.002)	1.20×10 ^-30^	128354	HbA1c	*HFE, HIST1H4A*
HbA1c	rs1800562	*HFE*	p.C279Y	A/G	0.051	-0.042 (0.003)	3.30×10 ^-47^	138093	HbA1c	*HFE, HIST1H4A*
FG	rs10305492	*GLP1R*	p.A316T	A/G	0.014	-0.08 (0.008)	2.37×10 ^-25^	129601	FG	*GLP1R*
HbA1c	rs35332062	*MLXIPL*	p.A358V	A/G	0.117	0.011 (0.002)	6.18×10 ^-9^	144042	HbA1c	*MLXIPL*
HbA1c	rs3812316	*MLXIPL*	p.Q241H	G/C	0.112	0.012 (0.002)	2.15×10 ^-8^	108605	HbA1c	*MLXIPL*
FG	rs194524	*STEAP2*	p.R456Q	A/G	0.523	0.01 (0.002)	7.65×10 ^-8^	129629	FG, T2D, RG	*STEAP2-AS1*
HbA1c	rs34664882	*ANK1*	p.A1503V	A/G	0.026	-0.049 (0.004)	2.43×10 ^-39^	144034	HbA1c	*ANK1*
FG	rs13266634	*SLC30A8*	p.R276W	T/C	0.305	-0.029 (0.002)	1.63×10 ^-46^	129614	FG, HbA1c, T2D	*SLC30A8*
HbA1c	rs13266634	*SLC30A8*	p.R276W	T/C	0.300	-0.015 (0.001)	8.50×10 ^-28^	143982	FG, HbA1c, T2D	*SLC30A8*
HbA1c	rs11557154	*DCAF12*	p.R113Q	T/C	0.138	-0.009 (0.002)	1.70×10 ^-7^	144045	T2D, HbA1c	*Mahajan 2022 from CMD KP*
FG	rs17853166	*IKBKAP*	p.S251G	C/T	0.026	-0.037 (0.006)	4.82×10 ^-11^	129640	FG	*IKBKAP*
HbA1c	rs60980157	*GPSM1*	p.S391L	T/C	0.246	-0.013 (0.002)	6.71×10 ^-17^	118824	FG, T2D	*GPSM1*
FG	rs60980157	*GPSM1*	p.S391L	T/C	0.254	-0.014 (0.002)	2.35×10 ^-9^	110915	FG, T2D	*GPSM1*
HbA1c	rs906220	*HK1*	p.H7R	G/A	0.916	0.025 (0.003)	2.16×10 ^-21^	94970	HbA1c	*HK1*
FG	rs701865	*PDE6C*	p.S270T	A/T	0.366	-0.01 (0.002)	1.14×10 ^-7^	118580	FG, RG	*PDE6C*
HbA1c	rs61732434	*OR51V1*	p.S161N	T/C	0.008	-0.052 (0.009)	1.75×10 ^-8^	127507	HbA1c	*HBB*
HbA1c	rs415895	*SWAP70*	p.Q447E	G/C	0.641	-0.013 (0.001)	1.15×10 ^-21^	138028	HbA1c	*SWAP70*
HbA1c	rs117706710	*AMPD3*	p.V311L	T/G	0.009	0.037 (0.006)	2.32×10 ^-10^	144048	HbA1c	*AMPD3*
FG	rs2167079	*ACP2*	p.R29Q	T/C	0.340	0.016 (0.002)	7.99×10 ^-15^	129580	FG	*MADD*
HbA1c	rs35233100	*MADD*	p.R766X	T/C	0.055	-0.015 (0.003)	1.13×10 ^-8^	144034	FG	*MADD*
FG	rs35233100	*MADD*	p.R766X	T/C	0.054	-0.029 (0.004)	1.46×10 ^-12^	126231	FG	*MADD*
FG	rs56200889	*ARAP1*	p.Q802E	C/G	0.270	-0.016 (0.002)	1.79×10 ^-14^	122674	FG	*ARAP1*
HbA1c	rs643788	*DPAGT1*	p.I393V	C/T	0.425	-0.006 (0.001)	1.77×10 ^-7^	144009	HbA1c	*C2CD2L*
FI ^1^	rs145878042	*RAPGEF3*	p.L300P	G/A	0.011	-0.054 (0.01)	1.15×10 ^-7^	91485	FI/HbA1c	*HDAC7/ PFKM*
HbA1c	rs2732481	*ZNF641*	p.Q363P	G/T	0.315	-0.009 (0.001)	2.07×10 ^-11^	142280	HbA1c	*SENP1*
HbA1c	rs3184504	*SH2B3*	p.W262R	C/T	0.567	0.007 (0.001)	5.98×10 ^-8^	138551	HbA1c	*ATXN2*
2hGlu	rs1169288	*HNF1A*	p.I75L	C/A	0.345	0.06 (0.011)	7.90×10 ^-9^	44278	T2D, 2hGlu	*HNF1A*
HbA1c	COSM147717	*ATP11A*	p.M317V	G/A	0.748	0.009 (0.001)	3.77×10 ^-12^	144022	HbA1c	*ATP11A,TUBGCP3*
HbA1c	rs229587	*SPTB*	p.S439N	T/C	0.357	0.007 (0.001)	2.60×10 ^-8^	134780	HbA1c	*SPTB*
HbA1c	rs35097172	*SLC25A47*	splice region variant, 5’ UTR variant	T/C	0.216	-0.008 (0.002)	5.67×10 ^-8^	144028	FG	*SLC25A47*
2hGlu	rs3784634	*VPS13C*	p.R974K	T/C	0.540	-0.069 (0.011)	6.40×10 ^-10^	37217	2hGlu	*VPS13C/ C2CD4A/ C2CD4B*
HbA1c ^1^	rs3747481	*PRR14*	p.P359L	T/C	0.261	0.009 (0.002)	3.30×10 ^-8^	103338	HbA1c	*ITGAD*
HbA1c	rs201226914	*PIEZO1*	p.L939M	T/G	0.002	-0.159 (0.015)	4.42×10 ^-26^	144024	HbA1c	*CDT1,CYBA*
2hGlu	rs72839768	*DVL2*	p.T529I	A/G	0.020	0.197 (0.03)	4.10×10 ^-11^	57866	T2D, 2hGlu	*SLC16A13*
HbA1c	rs2748427	*TMC6*	p.W125R	G/A	0.233	0.027 (0.002)	8.56×10 ^-70^	132326	HbA1c	*TMC6*
HbA1c	rs7225887	*B3GNTL1*	p.A163T	T/C	0.211	-0.015 (0.002)	5.73×10 ^-22^	125749	HbA1c	*FN3KRP, FN3K*
HbA1c	rs35413309	*RGS9BP*	p.A223V	T/C	0.030	-0.02 (0.004)	1.42×10 ^-8^	141598	HbA1c	*PDCD5*
2hGlu	rs1800437	*GIPR*	p.E318Q	C/G	0.217	0.103 (0.011)	2.59×10 ^-23^	56252	2hGlu	*GIPR*
FG	rs17265513	*ZHX3*	p.N310S	C/T	0.188	0.016 (0.002)	2.59×10 ^-10^	126253	FG	*ZHX3*
HbA1c	rs855791	*TMPRSS6*	V727A	G/A	0.577	-0.019 (0.001)	9.46×10 ^-51^	143907	HbA1c	*TMPRSS6*
FG	rs15943	*MAP3K15*	p.Q1083E	C/G	0.005	-0.084 (0.014)	2.83×10 ^-9^	67004	glucose	*PDHA1/MAP3K15*
FG	rs56381411	*MAP3K15*	p.G670S	T/C	0.005	-0.085 (0.013)	1.51×10 ^-11^	62319	glucose	*PDHA1/MAP3K15*
HbA1c	rs2229241	*RENBP*	splice acceptor variant	C/T	0.012	-0.123 (0.007)	1.14×10 ^-62^	95622	HbA1c	*G6PD*
HbA1c	rs1050828	*G6PD*	p.V68M	T/C	0.007	-0.334 (0.008)	7.41×10 ^-322^	112209	HbA1c	*G6PD*

**Table 2.** Gene-based results from broad (NSbroad mask) and strict (NSstrict mask) analyses. Genes in bold are newly discovered from this effort. N var: total number of variants in that gene-based analysis; *P*_burden_: p-value from burden test which assumes all variants have the same direction of effect; *P*_SKAT_: p-value from SKAT test which allows for different directions of effect between variants. The lowest p-value is highlighted in bold.

Trait	Gene	NSbroad mask			NSstrict mask		
		N var	*P _burden_*	*P _SKAT_*	N var	*P _burden_*	*P _SKAT_*
FG	***G6PC***	9	**1.41×10 ^-6^**	1.32×10 ^-5^	3	1.41×10 ^-3^	7.43×10 ^-4^
FI	***G6PC***	8	**1.62×10 ^-6^**	8.58×10 ^-6^	3	1.85×10 ^-3^	7.80×10 ^-3^
HbA1c	***TF***	10	**2.15×10 ^-6^**	5.98×10 ^-3^	3	5.48×10 ^-2^	5.48×10 ^-2^
FG	*MAP3K15*	18	**1.86×10 ^-25^**	1.07×10 ^-18^	7	1.34×10 ^-14^	4.01×10 ^-11^
HbA1c	*MAP3K15*	18	**1.27×10 ^-7^**	1.53×10 ^-04^	7	2.65×10 ^-4^	9.46×10 ^-3^
FG	*G6PC2*	18	4.09×10 ^-67^	5.38×10 ^-58^	7	**7.8×10 ^-69^**	3.83×10 ^-56^
HbA1c	*G6PC2*	18	6.18×10 ^-30^	4.65×10 ^-27^	7	**1.04×10 ^-31^**	1.92×10 ^-26^
FG	*SLC30A8*	13	5.69×10 ^-4^	**6.42×10 ^-11^**	7	6.55×10 ^-11^	3.74×10 ^-10^
HbA1c	*SLC30A8*	12	7.20×10 ^-8^	2.18×10 ^-5^	6	**5.66×10 ^-8^**	3.22×10 ^-6^
FG	*VPS13C*	52	9.66×10 ^-6^	**3.73×10 ^-7^**	26	1.27×10 ^-5^	1.44×10 ^-5^

### Identification of single-variant associations

Our single variant analyses identified 62 distinct coding variant associations at 58 genes associated with at least one of the glycemic traits at exome-wide significance ( *P* <2.2 × 10 ^-7^) ( Table 1). Of these, four variants at three genes represented novel associations. These included a missense (rs1983210, p.E1365D) and a splice region variant (rs3183099) in *OBSL1* associated with FI, another missense variant (rs1886686, p.G12A) in *WDR78* associated with FG, and a missense variant (rs31244, p.D543N) in *SV2C* associated with HbA1c ( Table 1). In addition, the missense variant (rs146886108, p.R187Q) in *ANKH* which was previously associated with T2D was associated for the first time with FG.

### Identification of gene-based associations

Our gene-based analyses identified six genes associated with glycemic traits, including *G6PC* and *TF* that had not been associated with glycemic traits before ( Table 2 and Supplementary Table S2 ^11^). These findings provide new hypotheses for downstream follow-up studies in the context of glycemic trait biology. *G6PC*, encoding glucose-6-phosphatase, is associated with FG and FI and is a homolog of *G6PC2. G6PC2 is* an established effector gene at a GWAS locus which contains multiple coding variants known to influence FG and HbA1c but not FI levels ^4, 5, 28– 30^. Loss-of-function variants at *SLC30A8* have been previously associated with reduced risk of T2D ^31– 33^, while *VPS13C* maps to the *VPS13C*/ *C2CD4A*/ *C2CD4B* T2D risk locus. Follow-up studies at this locus have with varying levels of evidence suggested *C2CD4A*, encoding a calcium-dependent nuclear protein, as the causal gene for T2D through its potential role in the pancreatic islets ^34– 37^. We found evidence of association at *MAP3K15* with reduced levels of FG and HbA1c ( Table 2 and Supplementary Table S2 ^11^), which is consistent with recent reports of the gene’s association with reduced levels of HbA1c and glucose, and reduced T2D risk ^6, 38^. Our analyses also detected *TF* (encoding transferrin) as a novel gene-based association signal associated with HbA1c but not any of the other glycemic traits, consistent with the role of the protein as the main iron carrier in the blood ( Table 2 and Supplementary Table S2 ^11^).

### Pathway analyses identify relevant gene sets regulating glycemia

Next, we used our coding variant association results to identify pathways enriched for glycemic trait associations, and to subsequently determine the extent to which different associations within the same trait implicate the same or similar pathways (as indicated by the functional connectivity of the network). To do this we used GeneMANIA network analysis ^39^, which takes a query list of genes and finds functionally similar genes based on large, publicly available biological datasets, that include protein-protein, protein-DNA and genetic interactions, pathways, protein domains, protein and gene expression data. GeneMANIA taps on updated versions of these databases for its core and network analyses, to identify related genes of known functions based on our input list of genes. To increase power to connect genes in a network, we considered all genes harboring non-synonymous variants that reached *P* <1 × 10 ^-5^ (Supplementary Table S3 ^11^) for any of the four glycemic traits in our study and mapped the most significant non-synonymous variant at each locus to the respective gene (totaling 121 associations across all traits) (Methods). A high degree of connectivity was observed within the HbA1c network, with enrichment of processes related to blood cell biology such as porphyrin metabolism, erythrocyte homeostasis and iron transport ( Figure 1A and Supplementary Table S4 ^11^). In comparison, the network generated from FG-associated genes captured several processes known to contribute to glucose regulation and islet function, including insulin secretion, zinc transport and fatty acid metabolism ( Figure 1B and Supplementary Table S4 ^11^). Given that there were fewer genes associated with FI and 2hGlu, we were less powered to draw meaningful insights from the enriched pathways in those traits (Supplementary Figure S1 and Supplementary Table S4 ^11^).

We also performed gene set enrichment analysis (GSEA) using EC-DEPICT ^22, 23^ (Methods). The primary innovation of EC-DEPICT is the use of 14,462 gene sets extended based on large-scale co-expression data ^21, 24^. These gene sets take the form of z-scores, where higher z-scores indicate a stronger prediction that a given gene is a member of a gene set. To reduce some of the redundancy in the gene sets (many of which are strongly correlated with one another), we clustered them into 1,396 “meta-gene sets” using affinity propagation clustering ^25^. These meta-gene sets are used to simplify visualizations and aid interpretation of results. As before, we considered all loci with variants that reached *P* <1 × 10 ^-5^ (Supplementary Table S3 ^11^) for any of the four glycemic traits for defining input genes (Methods). When looking across all traits combined, we found 234 significant gene sets in 86 meta-gene sets with false discovery rate (FDR) of <0.05 (Supplementary Table S5A, Supplementary Figure S2A ^11^). As expected, we observed a strong enrichment of insulin- and glucose-related gene sets, as well as hormone secretion and cytoplasmic vesicle gene sets (in keeping with pancreatic beta cell insulin vesicle release). In agreement with the GeneMANIA network analyses, we also noted a particularly strong enrichment for blood-related pathways represented by gene sets such as erythrocyte differentiation and heme metabolic process, which was primarily driven by HbA1c-associated variants. This was likely because HbA1c levels are influenced not only by glycation but also by blood cell turnover rate ^1, 40, 41^. To disentangle blood cell turnover from effects due to glycation, we repeated the analysis excluding variants that were significantly associated with HbA1c only and found 128 significant gene sets in 53 meta-gene sets (FDR <0.05) ( Figure 1C, Supplementary Table S5B, Supplementary Figure S2B ^11^). Indeed, we noted that majority of the gene sets now implicated pathways relevant to the pancreatic islets and metabolic tissues, such as “abnormal glucose homeostasis”, “peptide hormone secretion”, “Maturity Onset Diabetes of the Young”, and multiple pathways involved in the regulation of glycogen, incretins, and carbohydrate metabolism, that were also seen in the FG only analysis ( Figure 1D, Supplementary Table S5D, Supplementary Figure S2D ^11^).

We also analyzed each of the four traits separately, to reveal trait-specific enriched gene sets (Supplementary Table S5, Supplementary Figure S2C-E, Supplementary Figure S3C-D ^11^, Methods). Overall, our network and pathway enrichment analyses provide insight into the biology underlying each glycemic trait and may facilitate the prioritization of specific genes or pathways across multiple different phenotypes.

## Discussion

Here we have described large scale meta-analyses results for coding variant and gene-based associations for four glycemic traits, FG, FI, HbA1c and 2Glu, and the downstream pathways and networks that are regulated by the associated genes. Our results identified three genes with novel single-variant associations with glycemic traits *OBSL1* (FI), *WDR78* (FG) and *SVC2* (HbA1c). *OBSL1* encodes a cytoskeletal protein related to obscurin, mutations in which have been shown to lead to an autosomal recessive primordial growth disorder (OMIM: 612921). Loss of OBSL1 leads to downregulation of CUL7, a protein known to interact with IRS-1, downstream of the insulin receptor signaling pathway ^42^. *WDR78* encodes a WD repeat-containing protein 78, the same variant rs1886686-C has been previously associated with a decrease in systolic blood pressure ^43^. However, none of the *OBSL1* (rs1983210, b = -0.018, p = 1.20 x 10 ^-4^, N = 144,114; rs3183099, b = -0.019, p = 1.36 x 10 ^-4^, N = 125,397) or *WDR78* (rs1886686, b = -0.017, p = 3.83 x 10 ^-5^, N = 164,878) variants we detected here reached exome-wide significance in our recent large multi-ancestry study ^1^. This, despite larger sample sizes and good genotype quality (info >0.8 for each of the variants for the majority of cohorts), suggesting caution in the interpretation of these findings, and the need for additional datasets testing these associations. The final variant, p.D543N in *SV2C,* was associated with HbA1c with p = 5.5 x 10 ^-5^ in the European meta-analysis ^1^, and with p = 1.37 x 10 ^-12^ in UK biobank ^44^. A second missense variant at this gene, p.T482S, is also strongly associated with HbA1c (p = 1.9 x 10 ^-16^) and with red blood cell distribution width in UK biobank (p = 3.3 x 10 ^-11^) ^44^, and with mean corpuscular volume (p = 3 x 10 ^-11^) ^45^. Given that variation in red blood cell traits can influence HbA1c levels ^1, 41^, associations between these missense variants suggest *SV2C* as the likely effector gene at this locus. Also, the absence of evidence for association between this gene and other glycemic traits suggests its effect on HbA1c is independent of glycemia.

The novel gene-based association of *G6PC* with FG and FI was notable. Homozygous inactivating alleles in *G6PC*, including both p.R83C and p.Q347X which are contained in our gene-based association (Table S2), are known to give rise to glycogen storage disease type 1a (GSD1a). GSD1a is a rare autosomal recessive metabolic disorder ^46, 47^, but this is the first time that rare coding variants in *G6PC* have been shown to influence FG and FI levels in normoglycemic individuals. The other novel gene-based association was between *TF* and HbA1c. TF encodes transferrin, an iron-binding transport protein that circulates at high levels in blood plasma as an important biological carrier of iron. Dysregulation of iron concentrations due to reduced transferrin levels or function could affect the measurement of HbA1c independently of glycemia ^48^. The presence of multiple coding variants within TF associated with red blood cell traits in UK biobank ^44^ lends additional support to this hypothesis.

Overall, our network and pathway analyses were highly concordant with each other and with other published data identifying processes related to glucose regulation and islet function, including insulin secretion and zinc transport associated with FG loci, and red blood cell biology processes amongst HbA1c associated loci ^1^. The FG network revealed linking nodes (that are not among the association signals) with known links to glucose homeostasis and diabetes, such as *GCK* (encoding the beta cell glucose sensor glucokinase), *GCG* (encoding the peptide hormone glucagon secreted by the alpha cells of the pancreas) and *GIP* (encoding the incretin hormone gastric inhibitory polypeptide). Notably, lipid related pathways associated with fasting glucose. One gene within the FG cluster for lipid-related pathways is *CERS2*, which encodes ceramide synthase 2, an enzyme known to be associated with the sphingolipid biosynthetic process ( Figure 1B, Supplementary Table S3 ^11^). Although *CERS2* is only nominally associated with FG and is significantly associated with HbA1c (rs267738: *P*_FG_ = 3.54 × 10 ^-7^; *P*_HbA1c_ = 6.96 × 10 ^-10^), it does not cluster together with any HbA1c-enriched pathway, suggesting that *CERS2* is regulating FG and HbA1c indirectly through its role in lipid metabolism.

## Conclusions

In conclusion, our results provided novel glycemic trait associations and highlighted pathways implicated in glycemic regulation. The summary statistics results are being made publicly available through various platforms so they can be harnessed with other data to aid effector gene identification.

## Data Availability

Open Science Framework (OSF): Underlying data for ‘Large-scale exome array summary statistics resources for glycemic traits to aid effector gene prioritization’, https://doi.org/10.17605/OSF.IO/K6W3B ^11^ This project contains the following underlying data: Table S1: Supplementary Table S1 – Cohort characteristics, genotyping and quality control (QC), glucose, insulin, 2hGlu and HbA1c analyses and covariates. Table S2: Supplementary Table S2 - Full gene-based results including all variants included in the masks, for both novel and previously-established genes Table S3: Supplementary Table S3 - All variants associated with FG, FI, HbA1c and/or 2hGlu in our analyses with P<10-5 Table S4: Supplementary Table S4 - Gene Set Enrichment Analysis by GeneMANIA network analysis showing enriched GO terms and Reactome pathways in the network for (A) HbA1c; (B) FG; (C) FI; (D) 2hGlu Table S5: Supplementary Table S5 - EC-DEPICT results Figure S1: Supplementary Figure S1 – GeneMANIA network analysis results Figure S2: Supplementary Figure S2 – EC-DEPICT results (UKBB permutations) Figure S3: Supplementary Figure S3 - EC-DEPICT results (Swedish permutations) Data are available under the terms of the Creative Commons Attribution 4.0 International license (CC-BY 4.0) GWAS Catalog: meta-analysis summary statistics of 2-hour glucose in African American ancestry. MAGICExome_2hGlu_AFR.tsv.gz, study accession number GCST90256400. https://identifiers.org/gcst:GCST90256400 GWAS Catalog: meta-analysis summary statistics of 2-hour glucose in European ancestry. MAGICExome_2hGlu_EUR.tsv.gz, study accession number GCST90256401. https://identifiers.org/gcst:GCST90256401 GWAS Catalog: multi-ancestry meta-analysis summary statistics of 2 hour glucose. MAGICExome_2hGlu_ALL.tsv.gz, study accession number GCST90256402. https://identifiers.org/gcst:GCST90256402 GWAS Catalog: meta-analysis summary statistics of fasting glucose in African American ancestry. MAGICExome_FG_AFR.tsv.gz, study accession number GCST90256403. https://identifiers.org/gcst:GCST90256403 GWAS Catalog: meta-analysis summary statistics of fasting glucose in East Asian ancestry. MAGICExome_FG_EAS.tsv.gz, study accession number GCST90256404. https://identifiers.org/gcst:GCST90256404 GWAS Catalog: meta-analysis summary statistics of fasting glucose in European ancestry. MAGICExome_FG_EUR.tsv.gz, study accession number GCST90256405. https://identifiers.org/gcst:GCST90256405 GWAS Catalog: meta-analysis summary statistics of fasting glucose in Hispanic ancestry. MAGICExome_FG_HISP.tsv.gz, study accession number GCST90256406. https://identifiers.org/gcst:GCST90256406 GWAS Catalog: meta-analysis summary statistics of fasting glucose in South Asian ancestry. MAGICExome_FG_SAS.tsv.gz, study accession number GCST90256407. https://identifiers.org/gcst:GCST90256407 GWAS Catalog: multi-ancestry meta-analysis summary statistics of fasting glucose. MAGICExome_FG_ALL.tsv.gz, study accession number GCST90256408. https://identifiers.org/gcst:GCST90256408 GWAS Catalog: meta-analysis summary statistics of fasting insulin in African American ancestry. MAGICExome_FI_AFR.tsv.gz, study accession number GCST90256409. https://identifiers.org/gcst:GCST90256409 GWAS Catalog: meta-analysis summary statistics of fasting insulin in East Asian ancestry. MAGICExome_FI_EAS.tsv.gz, study accession number GCST90256410. https://identifiers.org/gcst:GCST90256410 GWAS Catalog: meta-analysis summary statistics of fasting insulin in European ancestry. MAGICExome_FI_EUR.tsv.gz, study accession number GCST90256411. https://identifiers.org/gcst:GCST90256411 GWAS Catalog: meta-analysis summary statistics of fasting insulin in Hispanic ancestry. MAGICExome_FI_HISP.tsv.gz, study accession number GCST90256412. https://identifiers.org/gcst:GCST90256412 GWAS Catalog: meta-analysis summary statistics of fasting insulin in South Asian ancestry. MAGICExome_FI_SAS.tsv.gz, study accession number GCST90256413. https://identifiers.org/gcst:GCST90256413 GWAS Catalog: multi-ancestry meta-analysis summary statistics of fasting insulin. MAGICExome_FI_ALL.tsv.gz, study accession number GCST90256414. https://identifiers.org/gcst:GCST90256414 GWAS Catalog: meta-analysis summary statistics of HbA1c in African American ancestry. MAGICExome_HbA1c_AFR.tsv.gz, study accession number GCST90256415. https://identifiers.org/gcst:GCST90256415 GWAS Catalog: meta-analysis summary statistics of HbA1c in East Asian ancestry. MAGICExome_HbA1c_EAS.tsv.gz, study accession number GCST90256416. https://identifiers.org/gcst:GCST90256416 GWAS Catalog: meta-analysis summary statistics of HbA1c in European ancestry. MAGICExome_HbA1c_EUR.tsv.gz, study accession number GCST90256417. https://identifiers.org/gcst:GCST90256417 GWAS Catalog: meta-analysis summary statistics of HbA1c in Hispanic ancestry. MAGICExome_HbA1c_HISP.tsv.gz, study accession number GCST90256418. https://identifiers.org/gcst:GCST90256418 GWAS Catalog: meta-analysis summary statistics of HbA1c in South Asian ancestry. MAGICExome_HbA1c_SAS.tsv.gz, study accession number GCST90256419. https://identifiers.org/gcst:GCST90256419 GWAS Catalog: multi-ancestry meta-analysis summary statistics of HbA1c. MAGICExome_HbA1c_ALL.tsv.gz, study accession number GCST90256420. https://identifiers.org/gcst:GCST90256420 These data are also available from https://magicinvestigators.org/downloads/
