# Supplementary material for: Large-scale exome array summary statistics resources for glycemic traits to aid effector gene prioritization
Source: Wellcome Open Res. 2023 Oct 20;8:483. [Version 1] doi: 10.12688/wellcomeopenres.18754.1 (PMC11399760; doi:10.12688/wellcomeopenres.18754.1)
Supplement: Peer review history

# Reviewer report: Reviewer response for version 1

**DOI:** 10.21956/wellcomeopenres.20795.r96202

**Published:** 2024-09-13

**Authors:** Eiji Kutoh

**Affiliations:** 1 Higashitotsuka Memorial Hospital, Yokohama, Japan; 2 Gyoda General Hospital, Saitama, Japan

I have completed the review of the research paper titled **“Large-scale exome array summary statistics resources for glycemic traits to aid effector gene prioritization”** by Dr. Willems et al. Below are my comments:

 -Overall, the paper is well-written and provides a clear presentation of the background, methods, results, and conclusions. However, there are several points that I would like to address:

****

 -The main conclusions are clear, but their reproducibility might be questionable. The authors could strengthen their findings by validating the results using alternative approaches, preferably not relying solely on in silico methods but incorporating real experiments, such as molecular and cellular biology techniques.

****

 -There are too many authors listed, and it is unclear “who did what.” Authorship could be perceived as casual, with individuals included without clear contributions. However, I do not intend to intervene on this matter.

****

 -Are there any potential biases or confounding factors that could have influenced the results? The inclusion and exclusion criteria might introduce selection bias, especially given the narrowly defined population.

****

 -While the study includes participants from various ancestral groups, it is unclear how these individuals were selected or why the study predominantly focuses on certain ethnic groups (85% European). This could introduce bias and raises questions about the robustness of the findings in non-European populations. Will certain associations be more detectable in one group over another? What challenges arise from the smaller representation of other ethnicities? The generalizability of the findings is thus questionable, especially regarding whether ancestry might affect the genetic associations.

****

 -The justification for using exome arrays is unclear. Are there specific advantages of exome arrays compared to whole-exome sequencing? This method is not considered cutting-edge technology.

****

 -The descriptions of phenotypes are somewhat vague. While the metrics for FG, FI, 2hGlu, and HbA1c are provided, there is little detail on how these were measured across different cohorts. Was there any standardization across cohorts?

****

 -The distance-based clumping method for defining loci (500 kb apart) lacks explanation. Why was this particular threshold chosen? Could it exclude significant associations that are closer together?

****

 -What are the clinical interpretations and implications of these results? This aspect seems to be missing. The paper heavily emphasizes in silico data (statistical and computational findings), but more context is needed regarding the physiological and biological significance of the identified gene sets for glycemic traits. For instance, the results mention variants associated with traits but do not thoroughly discuss the clinical relevance or potential functional implications of these variants. How might the novel missense variant rs146886108 in ANKH, for example, influence FG or T2DM risk?

****

 -The exclusion of individuals with diabetes is mentioned, but the rationale could be elaborated upon. Could this exclusion introduce bias? Does it ensure that the identified associations are specific to glycemic traits in non-diabetic individuals?

****

 -The authors report identifying 62 distinct coding variant associations at 58 genes with exome-wide significance. However, there is little detail on the methods used to control for false positives beyond the Bonferroni correction threshold of P<2.2×10−7.

****

 -The association with HbA1c is considered significant, but there is insufficient discussion about potential confounding factors, such as the influence of red blood cell (RBC) traits on HbA1c. I would suggest softening the interpretation of SV2C as an effector gene, given the complex relationship between RBC traits and HbA1c. Experienced clinicians in this field would likely agree that it is not appropriate to base conclusions about glycemic control solely on HbA1c levels.

Is the work clearly and accurately presented and does it cite the current literature?

Yes

If applicable, is the statistical analysis and its interpretation appropriate?

Partly

Are all the source data underlying the results available to ensure full reproducibility?

Yes

Is the study design appropriate and is the work technically sound?

Partly

Are the conclusions drawn adequately supported by the results?

Yes

Are sufficient details of methods and analysis provided to allow replication by others?

Partly

Reviewer Expertise:

diabetology, molecular endocrinology, molecular and cellular biology

I confirm that I have read this submission and believe that I have an appropriate level of expertise to confirm that it is of an acceptable scientific standard.

# Reviewer report: Reviewer response for version 1

**DOI:** 10.21956/wellcomeopenres.20795.r69837

**Published:** 2024-01-22

**Authors:** Toshimasa Yamauchi

**Affiliations:** 1 Department of Diabetes and Metabolic Diseases, University of Tokyo Graduate School of Medicine, Tokyo, Japan

In the presented study, the authors have undertaken a comprehensive exome-wide association study (ExWAS) to identify genetic loci linked to glycemic traits. A characteristic aspect of this research is the utilization of ExWAS meta-analysis to examine variants in coding regions, an approach that complements previous studies emphasizing non-coding variants. By analyzing data from a large participant pool, predominantly of European ancestry, the study pinpoints single coding variants and gene-based associations that could act as potential effector genes for glycemic traits such as glycated hemoglobin (HbA1c), fasting glucose (FG), fasting insulin (FI), and 2hr glucose post-oral glucose challenge (2hGlu). Additionally, the study extends to pathway analyses, offering insights into gene sets regulating these traits. The transparency and accessibility of the study are beneficial to the research community, with summary statistics made available on their website and through the GWAS catalog.

 The study's methodology, while not novel, adheres to established conventions in the field, ensuring a foundation for their analyses. The discovery of a modest number of new loci and genes associated with glycemic traits, though limited in quantity, is worth reporting. These findings include the identification of four variants in three genes that represent novel associations, underscoring the potential for uncovering new pathways in glycemic regulation. The gene-based analysis further highlights six genes, including G6PC and TF, previously unlinked to glycemic traits.

 The findings, while not groundbreaking, are biologically consistent. The study reveals a notable enrichment in blood-related pathways, especially those involving erythrocyte differentiation and heme metabolic processes. This enrichment, predominantly driven by HbA1c-associated variants, underscores the multifaceted influence on HbA1c levels, which are affected by both glycation and blood cell turnover. By excluding variants solely associated with HbA1c, the researchers effectively isolated 128 significant gene sets within 53 meta-gene sets (FDR <0.05). This refinement of analysis illuminated pathways more directly related to pancreatic islet function and metabolic tissues. These pathways, including “abnormal glucose homeostasis”, “peptide hormone secretion”, and “Maturity Onset Diabetes of the Young”, as well as those involved in glycogen regulation, incretin function, and carbohydrate metabolism, align with findings from fasting glucose-only analyses. Such insights could enhance our understanding of the complex genetic and biological mechanisms underlying glycemic control.

Is the work clearly and accurately presented and does it cite the current literature?

Yes

If applicable, is the statistical analysis and its interpretation appropriate?

Yes

Are all the source data underlying the results available to ensure full reproducibility?

Yes

Is the study design appropriate and is the work technically sound?

Yes

Are the conclusions drawn adequately supported by the results?

Yes

Are sufficient details of methods and analysis provided to allow replication by others?

Yes

Reviewer Expertise:

Diabetes, Obesity, Genetics

I confirm that I have read this submission and believe that I have an appropriate level of expertise to confirm that it is of an acceptable scientific standard.
